# The influence of ceremonial settings on mystical and challenging experiences occasioned by ayahuasca: A survey among ritualistic and religious ayahuasca users

**DOI:** 10.3389/fpsyg.2022.857372

**Published:** 2022-07-15

**Authors:** Alexandre Augusto de Deus Pontual, Luís Fernando Tófoli, Clarissa Mendonça Corradi-Webster, Kim van Oorsouw, Alicia Raquel Osuna Delgado, Johannes G. Ramaekers

**Affiliations:** ^1^Department of Psychology, Faculty of Philosophy, Science and Letters, University of São Paulo, Ribeirão Preto, Brazil; ^2^Interdisciplinary Cooperation for Ayahuasca Research and Outreach (ICARO), Faculty of Medical Sciences, University of Campinas, Campinas, Brazil; ^3^Department of Clinical Psychological Science, Faculty of Psychology and Neuroscience, Maastricht University, Maastricht, Netherlands; ^4^Department of Neuropsychology and Psychopharmacology, Faculty of Psychology and Neuroscience, Maastricht University, Maastricht, Netherlands

**Keywords:** ayahuasca, setting, Santo Daime, UDV, neo-shamanic, mystical experiences, challenging experiences

## Abstract

Recent studies have recognized the importance of non-pharmacological factors such as setting to induce or promote mystical experiences or challenging experiences among ayahuasca users. This study aimed to evaluate the association between the setting in which ayahuasca is consumed and the intensity of mystical and challenging experiences considering three ayahuasca using traditions (União do Vegetal, Santo Daime and neo-shamanic groups). A cross-sectional analysis was performed on survey data collected online from 2,751 participants. The Setting Questionnaire for the Ayahuasca Experience (SQAE) was used to evaluate six dimensions of the setting characteristics. The Mystical Experience Questionnaire (MEQ) and the Challenging Experience Questionnaire (CEQ) were used to quantify the psychedelic experience. Ratings on every SQAE setting dimension were negatively correlated with ratings of the CEQ (*r* values between 0.21 and 0.36) for all ayahuasca using traditions. Regression analysis revealed that ratings on four SQAE dimensions (Social, Comfort, Infrastructure and Decoration) explained 41% of the variance in CEQ ratings. Associations between SQAE and MEQ ratings were relatively weak and confined to the dimensions Leadership and Comfort, explaining 14% of the variance in MEQ ratings. Ratings of Social context were higher among members of União do Vegetal compared to Santo Daime and neo-shamanic members. Ratings of Infrastructure, Comfort and Decoration were more consistently correlated with MEQ in the neoshamanic tradition compared to the other traditions. This study shows that the setting is an important moderator of a challenging experience under ayahuasca. Maximizing the quality of the setting in which ayahuasca is taken will reduce the chance of a challenging experience while contributing positively to a mystical experience. The present findings can be considered when designing rituals and the (social) environment of ayahuasca ceremonies, and indicate that the SQAE questionnaire can be employed to monitor the influence of ceremonial settings on the ayahuasca experience.

## Introduction

Ritualistic and religious use of psychedelic substances such as ayahuasca has been reported to occur in several South-American Indigenous cultures (Richards, 2008; Labate and Feeney, 2012; Carbonaro et al., 2016; Santos et al., 2017; Miller et al., 2019). Ayahuasca is a *N,N*-dimethyltryptamine (DMT) and β-carboline-rich decoction, traditionally made from the *Banisteriopsis caapi* vine with leaves of *Psychotria viridis* (Labate and Feeney, 2012). β-carboline alkaloids such as harmine, harmaline and tetrahydroharmine function as monoamine oxidase inhibitors (MAOI) allowing DMT to reach the central nervous system for a prolonged period of time. This leads to intense alterations in perception and sensory integration and the induction of an altered state of consciousness (McKenna et al., 1998; Riba et al., 2001; Tupper, 2008; Palhano-Fontes et al., 2015).

From the 1930s on, ayahuasca consumption expanded to syncretic religious groups. The most widespread today are União do Vegetal (UDV) and Santo Daime (McKenna et al., 1998; De Rios and Grob, 2005). These were mainly established in Brazilian urban centers, and by the end of the 1980s, they had also spread throughout Europe and North America (Metzner, 2014; Hartogsohn, 2021). During this period, neoshamanic rituals—influenced by Indigenous practices, Eastern religions and the previous syncretic ayahuasquero groups—also spread through Brazil and Europe (Metzner, 2014; Ivanescu and Berentzen, 2020). Consumption of Ayahuasca is allowed in Brazil for religious purposes, but in certain European countries (e.g., Italy, France) ayahuasca is a scheduled substance because of its assumed threat to public health (Labate and Feeney, 2012; Labate and Cavnar, 2014).

Within the UDV, Santo Daime and neoshamanic practices, some common elements can be observed, such as the concepts of healing (either physical or metaphysical) and spiritual growth. All three of these practices have leaders responsible for studying their doctrines and managing the rituals (Goulart, 2006; Hartogsohn, 2021). Doctrines are taught during the ayahuasca ceremony: in the Daime through lyrics (MacRae, 2004; Hartogsohn, 2021), in the UDV through question and answers and singing of *a cappella* chants (Goulart, 2006), and in neoshamanic rituals through chanting, hearing of songs or recordings and shamanic practices such as smoke blowing and herbal sprays (Gonzalez et al., 2021). In both UDV and Santo Daime, Christian symbolism predominates, with influences of other religious groups, such as spiritists and Afro-Brazilian religions. The latter have added elements of setting and symbolic repertoire, such as the belief in the presence of human and nature spirits (MacRae, 2004; Goulart, 2006). Traditional shamanism can be defined as a set of beliefs and ancestral rites, present in different cultures, each with its own particularities. This set of spiritual practices is centered around the figure of the shaman, who promotes altered states of consciousness in support of physical and emotional healing (Von Stuckrad, 2002; Townsend, 2004). The contact of traditional shamanism with Western culture from the 1970s promoted a series of modifications and syncretism that culminated in the neo-shamanic movement (Von Stuckrad, 2002). Besides the characteristics of traditional shamanism, Neo-shamanism includes elements from Eastern, Pagan, and Christian religions, among others (Townsend, 2004). Furthermore, it focuses on spiritual development from an individual logic, departing from the sense of collectivity that is characteristic of traditional shamanism (Von Stuckrad, 2002).

During these religious rituals, practices are performed aiming to facilitate the occurrence of mystical experiences and to prevent unpleasant or challenging experiences in the participants, such as singing religious music and choosing the space decoration and colors (Hartogsohn, 2021). It has also been noted that mystical experiences contain common elements that occur independent of their historical, cultural and religious context (Stace, 1960; MacLean et al., 2012; Roseman et al., 2018), such as a sense of unity, peace (Garcia-Romeu et al., 2015), sacredness, ineffability and joy, and the absence of time and space (MacLean et al., 2012). In addition, the consumption of psychedelic substances can also produce challenging experiences (Cohen, 1960; Barrett et al., 2016). Negative experiences may vary in intensity and duration and may result in transient outbursts of paranoia, sadness, anger, delirium, depersonalization, dissociation and confusion (Johnson et al., 2008; Barrett et al., 2016).

Specific causes and conditions that induce or promote mystical experiences (MacLean et al., 2012) or challenging experiences (Barrett et al., 2016) are not well understood. However, recent studies have recognized the importance of non-pharmacological factors, such as set and setting, as moderators of the behavioral and psychological effects of psychedelics (Carbonaro et al., 2016; dos Santos et al., 2016; Hartogsohn, 2016; Roseman et al., 2018). Set refers to the psychological state, intentions and expectations of individuals in psychedelic ceremonies, while setting refers to the context in which a ceremony occurs. The parameters of the setting can refer to sensory modes (e.g., auditory, musical; visual, tactile), the social environment (e.g., being alone or in a group, in nature or in a building, presence of a leader), the set of those present in a ceremony that surround an individual (Leary et al., 1963; Shewan et al., 2000; Hartogsohn, 2016, 2017; Haijen et al., 2018), or group dynamics and leadership (i.e., facilitators or hosts) before, during, and after ceremonies with psychedelics (Trope et al., 2019). Setting parameters may increase an individual’s sensitivity to a psychedelic experience and amplify its subjective effects (Carhart-Harris et al., 2018).

A tool to assess an individual’s perception of the setting during an ayahuasca ritual was recently developed by our group (Pontual et al., 2021). This Setting Questionnaire for the Ayahuasca Experience (SQAE) was developed from interviews with experienced ayahuasca users and validated using a large survey sample (Pontual et al., 2021) and allows the qualification of six major setting parameters (Leadership, Decoration, Infrastructure, Comfort, Instruction and Social Context).

Considering the attention given to the importance of ritualistic setting for the consumption of Ayahuasca, and its possible effects on mystical and challenging experiences, there is a need to analyze correlations between aspects of predominant ritualistic settings of ayahuasca with occurrences of mystical or challenging experiences. Therefore, the present study applied the SQAE aiming to evaluate the association between setting parameters during ayahuasca ceremonies and the occurrence of mystical and challenging experiences across three ayahuasca using traditions, i.e., UDV, and Santo Daime in religious Brazilian traditions and neo-shamanic practices in Western cultures.

## Materials and methods

### Participants and procedure

An online questionnaire was developed using Qualtrics. Data were collected in Brazil and the Netherlands. As a recruitment method for the Brazilian participants, invitations with a weblink to participate in the study were sent to members of different ayahuasca churches and neoshamanic communities. An institutional email was also sent by the head office of UDV church in Brazil to all registered members, encouraging them to participate in the study. For the data collection in the Netherlands invitations were sent to Dutch ayahuasca organizations that hosted retreats. The Dutch participants were not linked to an institution, like Daime or UDV.

Participants were asked to complete the questionnaire while thinking about their most recent ayahuasca experiences. Inclusion criteria for this study were: being at least 18 years old; having consumed ayahuasca for at least 6 months before the study. Exclusion criteria were: not having filled out all the fields in the survey; having completed it in less than 5 min (considered insufficient time); or giving the same answer to all the items (interpreted as invalid data). From a total of 3,472 participants, 2,751 were considered valid, 2,263 participants from Brazil and 488 from the Netherlands. Participants were also classified in one of three traditions; (Neo)shamanic (i.e., ayahuasca or an ayahuasca analogue was taken in a setting that was hosted by a shaman or in a shamanic ritualistic tradition), UDV, or Santo Daime.

### Instruments

#### Mystical experience questionnaire

Mystical Experience Scale (Mystical Experience Questionnaire – MEQ-30): is a self-report instrument, developed by Pahnke (1969) and validated by Barrett et al. (2015). Evidence of its validity for the Brazilian population was explored by Schenberg et al. (2017). Its reduced version contains 30 items. Participants were asked the degree to which they experienced a list of phenomena in their last ayahuasca experience ranging from (0 = not at all to 6 = extremely). Example items are: “experience of pure being and pure awareness” and “experience of unity with ultimate reality”. Evidence of its validity for the Brazilian population was explored by Schenberg et al. (2017), who demonstrated a satisfactory internal reliability index by Cronbach’s Alpha. The MEQ is a tool that has been widely used internationally to assess unique mystical experiences from the ingestion of psychedelics (MacLean et al., 2012).

#### Challenging experience questionnaire

The CEQ is a self-report instrument developed and validated in 2016 by Barret and Griffiths (Barrett et al., 2016). It aims to assess the phenomenological profile of psychedelic experiences characterized as challenging. The instrument is composed of 26 items and seven factors: sadness, fear, death, insanity, isolation, physical suffering, and paranoia. Participants were asked to rate the extent to which they experienced a list of phenomena in their most recent ayahuasca experience on a 5-point scale ranging for 0 = not at all, to 6 = extremely. Example items are “isolation or loneliness” and “fear that I may lose my mind or go insane.”

#### Setting questionnaire for the ayahuasca experience

The SQAE is a self-report instrument developed based on interviews with ayahuasca drinkers from different ritualistic backgrounds and levels of experience. It was developed by Pontual et al. (2021) and it aims to evaluate different elements of the setting of ritualistic ayahuasca consumption. It was validated among a sample of 2,994 Brazilian ayahuasca drinkers, presenting evidence of validity for its theoretical model composed of 6 dimensions with 28 Likert-type statements and supplementary descriptive information about the ritual. The six dimensions include: Social (i.e., how participants felt within the group, e.g., “I felt among equals in that group.” and “Looking at other people bothered me”), Leadership (i.e., how participants saw the organizers as experienced leaders or not; e.g., “The organizers showed themselves to be inexperienced” and “I entrusted all my concerns to the ritual support group”), Decoration (i.e., how they felt in the space; e.g. “The ceremony was held in a sufficiently open space”), Infrastructure (i.e., facilities and logistical details; e.g., “I found the bathroom to be inadequate”), Comfort (i.e., how comfortable the space was; e.g. “I missed a support for the spine, head or arms” and “The place where I was sitting/lying bothered me”) and Instructions (i.e., how they were given preparatory instructions about the event; e.g., “I was previously instructed about the whole ritual” and “There were times when I felt that there was a lack of instruction”). Scores on each item ranged from 1 (strongly disagree) to 5 (strongly agree), with higher scores meaning higher agreement on the importance of this element during the ritual (negative statements are reverse-scored). In addition, some questions are contained to explore additional ceremony features such as the use of live, recorded, religious, Indigenous and other types of music (participants indicate if those elements were present yes/no), presence of activities such as dancing or text reading, and how enjoyable they felt – answers starting at 0 (This activity was not performed.) and ranging from 1 (It was very unpleasant) to 6 (very pleasant) –, presence of natural elements (e.g., fire, animals, 0 = not present, 1 = present), and other possible disruptive stimuli (e.g., noise, insects 1 = did not bother me, 4 = bothered me a lot). It was originally developed in Brazilian Portuguese and English by Pontual et al. (2021) and translated to Dutch for the purpose of the present study.

### Data analysis

Statistical Package for the Social Sciences (SPSS), version 26 (SPSS, Chicago, IL, United States) was used to perform the descriptive and inferential analyses. The calculation of means, standard deviation, kurtosis, and skewness were used for the descriptive analyses. One-way ANOVAs were conducted to analyze differences in setting ratings between traditions. Bonferroni corrected, *post hoc* contrasts were conducted to assess differences between separate traditions. Two-tailed Pearson correlations were performed between SQAE ratings and ratings from MEQ and CEQ. Correlations stronger than *r* > 0.10 were considered relevant, and results with a *p* < 0.01 were considered significant. In addition, regression analyses were used to evaluate SQAE parameters as predictors of MEQ and CEQ ratings. In a second step, it was investigated whether traditions differed in their ratings and value of the different setting parameters. In the Supplementary Material, correlations and regressions on how the setting parameters were related to MEQ and CEQ experiences per tradition can be found.

## Results

### Participants

**Figure 1:**
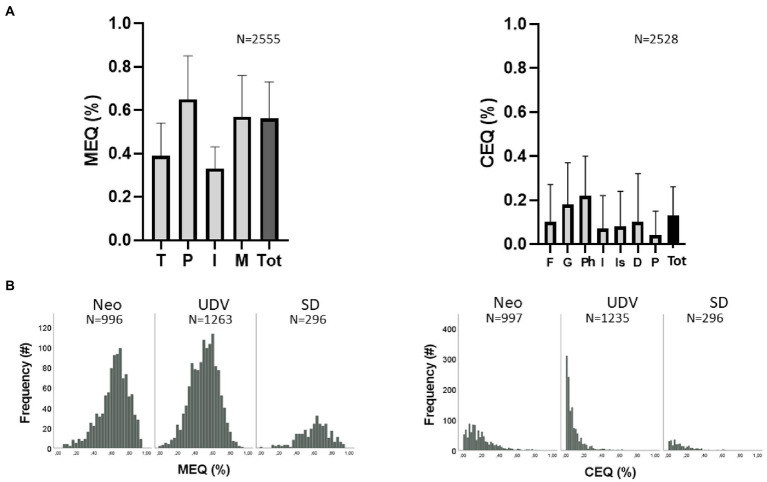
Mean (SD) subscale ratings and total scores of the Mystical Experience Questionnaire (MEQ) and Challenging Experience Questionnaire (CEQ) across the three ayahuasca conditions (panel **A**) and the frequency distribution of the total MEQ and CEQ scores per tradition (panel **B**). MEQ labels indicate transcendence (T), positive mood (P), ineffability (I), mysticism (M), and total score (Tot). CEQ labels indicate fear (F), grief (G), physical distress (P), insanity (I), isolation (Is), death (D) paranoia (P), and total score (Tot).

All participants that were included in the study had consumed ayahuasca within the last 6 months. The study included 47% males and 53% females. Eleven percent were between 18–23 years old, 19% aged 24–30, 30% aged 31–40, 34% aged 41–60, and 6% > 60 years old. Two percent of the participants completed basic education, 16% middle school and the majority followed some type of higher education (82%). Eight percent of the respondents were first time ayahuasca users, 10% drank less than five times, 13% drank between 5–20 times, 20% between 20–100 times, and 50% drank over 100 times ayahuasca (majority of those were members of the Santo Daime church or UDV). Of the 3,070 participants, 1,041 were categorized as (neo)shamanic, 1,413 as UDV, and 296 as SD. Figure 1 displays the average ratings of mystical (MEQ) and challenging (CEQ) experiences reported by participants on the different subscales, across traditions (see Figure 1).

### Setting questionnaire for the ayahuasca experience

**Figure 2:**
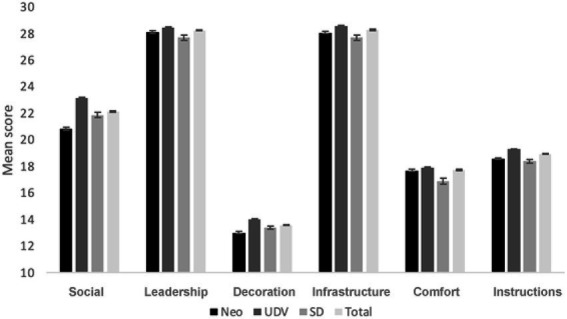
Mean Setting Questionnaire for the Ayahuasca Experience (SQAE) ratings. Specified for the entire sample (Total) and by tradition (Neo, UDV, SD). All between tradition *p*’s < 0.005, except Comfort – Neo vs. UDV, and Instructions – Neo vs. SD.

**Figure 3:**
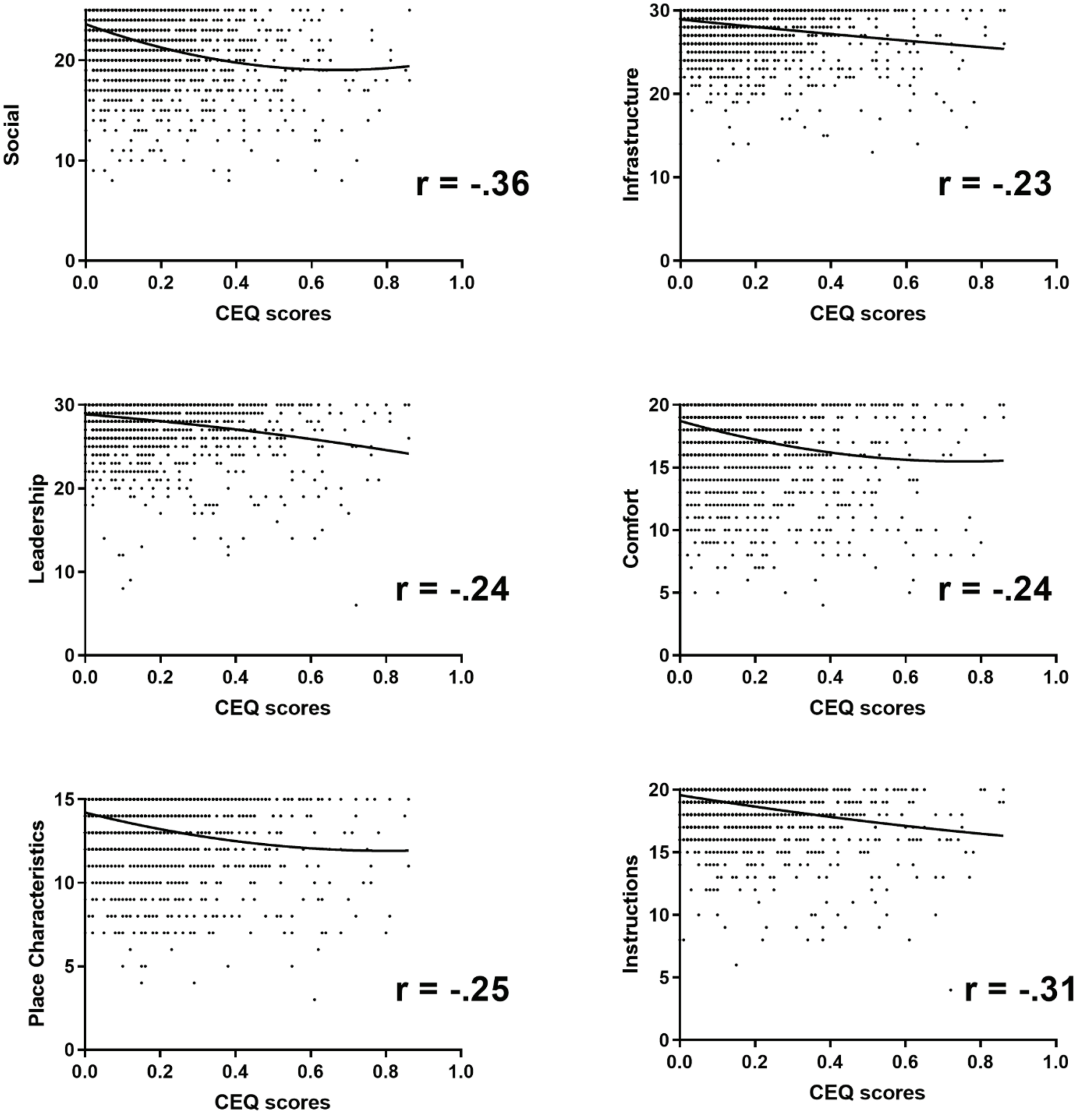
Correlations between SQAE scales (Social, Leadership, Decoration, Infrastructure, Comfort, and Instructions) and the CEQ total score.

Mean scores were calculated for all SQAE dimensions for the total group and for each tradition separately (see Figure 2). Their ratings at the Leadership and Infrastructure dimensions were highest, followed by Social aspects. Firstly, it was examined whether setting characteristics were related to reports of mystical and challenging experiences, as measured with MEQ and CEQ. Correlation analyses between the total MEQ and CEQ scores and setting characteristics indicated that for MEQ, only Leadership correlated positively with MEQ total score (*r* = 0.132, *p* < 0.001). All SQAE subscales correlated negatively with the level of self-reported challenging experiences (see Figure 3). That is, higher endorsement of the importance of all setting elements was associated with fewer self-reported challenges. Correlation between the presence of stimuli and natural elements and self-reported mystical and challenging experiences can be found in the Supplementary Material.

In a second step, using regression analyses, it was investigated whether certain setting elements predicted higher MEQ and CEQ scores. A linear regression analysis was conducted with MEQ total score as dependent variable and the six SQAE dimensions as independent variables. Stepwise backward elimination with a criterion of *p* < 0.05 resulted in a model in which “Comfort” and “Leadership” were predictive of MEQ scores. The model accounted for 14% of the variance. A similar procedure was followed for CEQ scores, resulting in the final model with the elements “Social,” “Decoration,” “Comfort,” and “Instructions” being predictive of CEQ ratings, together explaining 41% of the variance of CEQ ratings (see Tables 1 and 2).

**Table 1:** Summary of linear regression with Mystical Experience Questionnaire (MEQ) and Challenging Experience Questionnaire (CEQ) as dependent variables and the setting elements (social, leadership, decoration, infrastructure, comfort and instructions) as independent variables.

	*B*	*SE*	*β*	*t*	*p*
MEQ					
Comfort	0.002	0.001	0.041	1.960	0.050
Leadership	0.008	0.001	0.118	5.618	<0.001
CEQ					
Social	−0.010	0.001	−0.232	−10.81	<0.001
Decoration	−0.003	0.001	−0.047	−2.61	0.031
Comfort	−0.005	0.001	−0.111	−5.54	<0.001
Instructions	−0.010	0.002	−0.138	−6.34	>0.001

B, regression coefficient; SE, standard error; β, beta.

**Table 2:** Summary of linear regression by tradition [neo-shamanic, União do Vegetal (UDV) and SD] with MEQ and CEQ as dependent variables and the setting elements (social, leadership, decoration, infrastructure, comfort and instructions) as independent variables.

	*B*	*SE*	*β*	*t*	*p*
MEQ neo-shamanic					
Social	0.010	0.002	0.212	6.473	<0.001
Infrastructure	0.008	0.002	0.123	4.032	<0.001
UDV					
Social	0.005	0.002	0.086	2.731	0.006
Leadership	0.007	0.002	0.099	3.129	0.002
SD					
Stability	0.016	0.004	0.226	3.985	<0.001
CEQ neo-shamanic					
Social	−0.005	0.002	−0.115	−3.82	0.001
Leadership	−0.007	0.002	−0.140	−3.95	<0.001
Comfort	−0.005	0.002	−0.102	−3.04	0.002
UDV					
Social	−0.008	−0.001	−0.202	−6.662	<0.001
Decoration	−0.005	−0.002	−0.084	−2.775	0.006
Infrastructure	−0.003	−0.001	−0.062	−2.167	0.030
Comfort	−0.006	−0.001	−0.181	−6.164	<0.001
SD					
Social	−0.013	0.002	−0.329	−5.733	<0.001
Comfort	−0.008	0.002	−0.221	−3.854	<0.001

B, regression coefficient; SE, standard error; β, beta.

The second objective was to see whether setting elements differentially affected MEQ and CEQ ratings in the three ayahuasca using traditions. Figure 2 displays mean scores on the six main setting dimensions per tradition. Although all traditions rated Leadership and Infrastructure as most important setting elements, followed by Social, Instructions, Comfort and Decoration (see Figure 2), there were significant differences between traditions. One-way ANOVA showed that ratings at all SQAE dimensions significantly differed between traditions (all *F*’s > 10.48, all *p*’s < 0.001). The largest difference between traditions was found for the Social subscale (F2, 2,749 = 178.526, *p* < 0.001) with UDV rating this as more important than neo-shamanic and SD.

Separate correlations were calculated between ratings of the six setting dimensions and MEQ/CEQ ratings per tradition. As can be seen in Table 3, setting dimensions were more positively correlated to MEQ ratings in the neo-shamanic and Santo Daime traditions than in the UDV tradition. For CEQ ratings, in all traditions higher ratings of all setting dimensions were inversely related to CEQ ratings, albeit stronger in the religious traditions than in the neoshamanic groups.

**Table 3:** Correlations between the MEQ/CEQ and Setting Questionnaire for the Ayahuasca Experience (SQAE) elements (social, leadership, decoration, infrastructure, comfort and instructions) by tradition.

	MEQ			CEQ		
SQAE subscales	Neo	UDV	SD	Neo	UDV	SD
Social	0.260**	0.133**	0.186*	−0.202**	−0.325**	−0.427**
Leadership	0.192**	0.139**	0.218**	−0.227**	−0.220**	−0.314**
Decoration	0.170**		0.168*	−0.141**	−0.254**	−0.231**
Infrastructure	0.210**		0.124*	−0.187**	−0.208**	−0.312**
Comfort	0.176**			−0.191**	−0.304**	−0.367**
Instructions	0.188**	0.109**	0.226**	−0.293**	−0.205**	−0.311**

* *p* < 0.010 and

** *p* < 0.001.

The setting questionnaire also inquired about the presence of secondary elements such as music, noise and activities performed.

**Figure 4:**
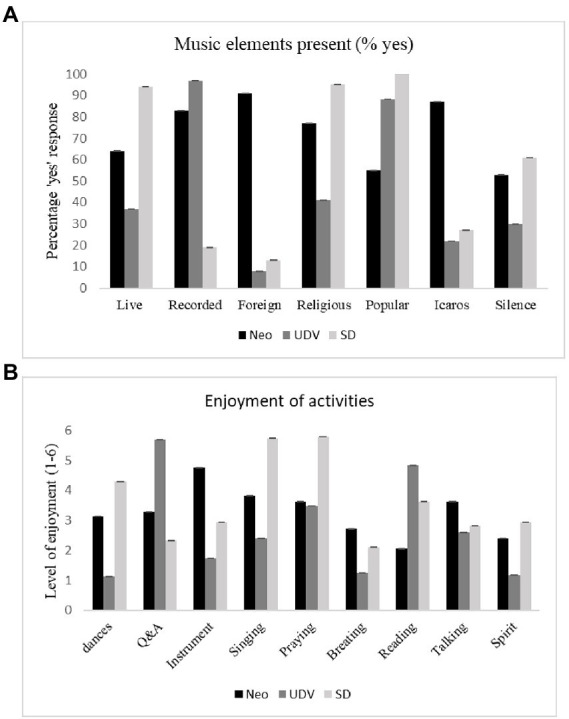
Differences between traditions in their rating of musical elements (presence yes/no, **A**) and enjoyment of activities during their most recent ayahuasca ritual (0 = not present 6 = enjoyed a lot, **B**).

Chi square analyses were conducted to see whether the presence of different music elements differed across traditions. Figure 4 gives the percentages of “yes” responses to the presence of different musical elements per tradition. For all elements, chi square tests were significant (all X2 > 1.762, all *p*’s < 0.001).

Across traditions music elements contributed to the mystical experience with the highest positive correlations existing between MEQ and music in a foreign language (*r* = 0.301, *p* < 0.001), religious music (*r* = 0.221, *p* < 0.001) and *icaros* (South-American traditional ayahuasca singing; *r* = 0.314, *p* < 0.001). When exploring differences between traditions, we found the above pattern for UDV but not for neo-shamanic groups and Santo Daime. That is, for neo-shamanic participants, only *icaros* were positively correlated to MEQ scores (*r* = 0.101, *p* = 0.001). For Santo Daime only silence was related to mystical experiences (*r* = 0.149, *p* = 0.010). Only for UDV, the presence of *icaros/*indigenous music obtained a higher correlation with both mystical and challenging experiences (*r* = 0.162, *p* < 0.001). It is noteworthy that in the case of the UDV that almost 100% of participants reported the presence of recorded music, a regular feature of UDV sessions. However, less than 50% reported religious music and less than 40% indicated the presence of live music, which probably indicates a flaw in the instrument in detecting the traditional *a cappella* UDV chants, named chamadas (“callings”), which are performed live and a key element in absolutely all UDV sessions. They are not understood and referred to as “music,” and the idea of “singing” does not apply to them (chamadas are “done”; not “sung”). Therefore, it is possible that many UDV members did not classify chamadas in any of the available options. The instrument should be corrected to include these chants in a more explicit way and data should be interpreted with caution.

Participants were also asked how much they enjoyed certain activities that were performed during the ceremonies or rituals in which they most recently drank ayahuasca. Figure 4B shows how much an activity was enjoyed in each of the traditions. Except for “praying” and “talking,” all traditions differed significantly in their enjoyment of the activities (all *F*’s > 66.52, all *p*’s < 0.001).

Only for the neo-shamanic tradition, dancing and praying were positively related to the level of mystical experiences (*r* = 0.234 and *r* = 0.268, respectively), not for the other traditions. In none of the traditions, activities were related to the level of challenging experiences.

## Discussion

The present study investigated the association between setting parameters during ayahuasca ceremonies and the occurrence of self-reported mystical and challenging experiences of ayahuasca users from UDV, Santo Daime and neoshamanic traditions, as well as whether setting ratings differed between the three ayahuasca traditions. Total scores in all SQAE subscales negatively correlated with total scores in the CEQ. Therefore, higher (quality) ratings of setting dimensions during the ceremonies were associated with a lower incidence of challenging experiences. A positive correlation was found between (quality) ratings of setting dimensions and ratings of mystical experience. Regression analyses including all denominations showed that higher ratings of feeling part of the social group (Social dimension), feeling physically comfortable, and at ease with the decorations of the space (Decoration dimension) and having had adequate instructions (Instructions) predicted fewer challenging experiences. Analyses per tradition showed some minor differences between traditions, with UDV having Infrastructure, and neo-shamanic having Leadership as one of their predictors. Adequate Leadership and Comfort were associated with higher self-reported levels of mystical experiences in the regression analyses including all denominations, but analyses per tradition showed discrepancy among traditions, with the Stability dimension being the sole predictor to show in the Santo Daime regression.

The influence of leadership on the occurrence of mystical experiences may be related to the feeling of being guided by a more advanced practitioner, which could increase the level of confidence in the experience. Clinical trials conducted with the use of psychedelics have suggested the importance of having someone with a non-judgmental posture caring for the participant and guiding their experience (Johnson et al., 2008; Carhart-Harris et al., 2018). Considering the Social dimension, feeling integrated with other participants within the ritual was found to be important for fewer challenging experiences. This may be related to a large part of the sample being composed of members of the UDV and Santo Daime, churches where the sense of community is reinforced (MacRae, 2004; Hartogsohn, 2017) and has also being reported as correlated to positive outcome of psychedelic rituals (Kettner et al., 2021). Similarly, the influence of decoration was also related to the prevention of challenging experiences, as found by Hartogsohn (2017), who cited the importance of space organization for the therapeutic potential with psychedelics. Decorations that conform to spiritual beliefs can also provide a sense of safety and wholeness.

The second objective was to assess whether the setting dimensions were rated differently between the three traditions. Ratings in all setting dimensions differed significantly between all traditions and were most apparent for ratings in the Social Dimension. Ratings in the Social Dimension were highest among UDV members, followed by those of Santo Daime, and lowest among followers of the neo-shamanic tradition. This finding can be explained by differences in social elements among each tradition. Santo Daime and UDV are religions in which their members attend weekly or fortnightly rituals (De Rios and Grob, 2005). Therefore, the social context of these traditions is associated with a strong sense of community. The promotion of other activities within the religion and outside the ritual itself also reinforces the creation of bonds. Apud (2015) and Townsend (2004) highlight the fact that in neo-shamanic settings it is common that motivations of visitors are either driven by curiosity or the need for psychological healing due to existential conflicts, using this as a psychotherapeutic device for specific problems and transformations in their daily lives. Therefore, a stronger focus on the individual experience is observed, independent of others who may be present at the ceremony. Accordingly, its social context relates more to the intention of a new experience, and less to the affiliation with a religious doctrine or community (Apud, 2015).

Infrastructure, Comfort and Decoration ratings were more consistently correlated with the MEQ in the neo-shamanic tradition compared to the religious traditions. In neoshamanic ceremonies participants usually have an individual experience (i.e., laying on their mattress) and there is little group interaction, in comparison with Santo Daime e UDV (Goulart, 2006; Hartogsohn, 2021). This may explain the neoshamanic emphasis on individual experience-oriented elements to induce mystical experiences, such as good site infrastructure and feeling comfortable. Conversely, within a Santo Daime or UDV ritual other factors (i.e., social) were reported to be more important. As shown in Figure 4, the presence of group activities such as singing and praying were rated higher among members of Santo Daime (see also MacRae, 2004), whereas questions and answers and reading were more valued among UDV members.

Regarding other elements in addition to the SQAE factors, music has already been described as an important element of the psychedelic consumption setting (Zinberg, 1984; Barrett et al., 2018; Carhart-Harris et al., 2018; Hartogsohn, 2021). Analysis of music elements revealed that MEQ ratings were positively correlated with music in foreign languages, religious music and *icaros*, across the three traditions. This association was most apparent in the UDV, whereas in the neo-shamanic tradition and Santo Daime correlations with MEQ were specific to *icaros* and silences, respectively. These differences may be related to the cultural load and meanings attributed to the musical element in each tradition. Regarding the neoshamanic tradition, *icaros* assume a central conducting role (Labate and Cavnar, 2014; Apud, 2015), and may be associated with an expression of visual effects (Labate and Cavnar, 2014). Apud (2015) points out that they can also serve as a psychological support, at times when the experience becomes chaotic. In Santo Daime, the songs (mostly with religious content) are encouraged to be sung by all participants, and acquire a significance that contributes to increase the closeness among them (Hartogsohn, 2021).

Likewise, the ratings of activities performed during the ayahuasca ceremonies significantly differed among the traditions. Only for the neo-shamanic tradition such activities, i.e., dancing and praying, were positively related to the level of mystical experiences, despite dancing and praying being core elements of the various religious traditions (MacRae, 2004; Hartogsohn, 2021). The fact that the participants of Santo Daime and UDV frequently attend rituals might cause these activities to be perceived as commonplace, decreasing their impact on the mystical experience.

The current data also comes with limitations. The majority of the sample consisted of UDV members and visitors of neo-shamanic ceremonies, with members of Santo Daime only comprising about 10% of the entire sample. Ratings of the latter group therefore might be less representative than those obtained from the other traditions. Furthermore, about 50% of the sample had drunk ayahuasca over 100 times, which may have affected their reports of the relevance of the setting parameters, with the perception of adverse effects possibly varying according to the participants’ length of experience (Durante et al., 2020).

Finally, correlations obtained in large datasets are easily inflated. Even very low correlations can achieve statistical significance with very large sample sizes, even in the absence of clinical relevance. The risk of overinterpreting the current findings was reduced by thresholding *p* and *r* values, however, it cannot be totally excluded.

This study shows that the setting seems to be an important moderator of mystical and challenging experiences during ayahuasca ceremonies, which is in agreement with the studies that reinforce this influence of the setting for the use of ayahuasca (Thomas et al., 2013; Hartogsohn, 2021) and other psychedelic substances (Hartogsohn, 2016; Carhart-Harris et al., 2018; Haijen et al., 2018). Maximizing the quality of the setting in which ayahuasca is consumed may increase the likelihood of a mystical experience and reduce the chance of a challenging experience. The present findings can be taken into account when designing the rituals and (social) environment of ayahuasca ceremonies, and indicate that the SQAE questionnaire can be employed to monitor the influence of ceremonial settings on the ayahuasca experience.

## Data availability statement

The raw data supporting the conclusions of this article will be made available by the authors, without undue reservation.

## Ethics statement

The studies involving human participants were reviewed and approved by Comitê de Ética em Pesquisa da Faculdade de Filosofia, Ciências e Letras de Ribeirão Preto – USP CAAE: 64130517.8.0000.5407. The patients/participants provided their written informed consent to participate in this study.

## Author contributions

AP, AD, KO, and JR devised the project, designed the study, and developed the theory. AP, AD, and KO performed data analysis. JR verified the analytical methods. All authors provided critical feedback and helped shape the research, analysis, and manuscript. All authors contributed to the article and approved the submitted version.

## Conflict of interest

The authors declare that the research was conducted in the absence of any commercial or financial relationships that could be construed as a potential conflict of interest.

## Publisher’s note

All claims expressed in this article are solely those of the authors and do not necessarily represent those of their affiliated organizations, or those of the publisher, the editors and the reviewers. Any product that may be evaluated in this article, or claim that may be made by its manufacturer, is not guaranteed or endorsed by the publisher.

## Supplementary materials

The Supplementary Materials for this article can be found online at: https://www.frontiersin.org/articles/10.3389/fpsyg.2022.857372/full#supplementary-material
