# Supplementary material for: The influence of ceremonial settings on mystical and challenging experiences occasioned by ayahuasca: A survey among ritualistic and religious ayahuasca users
Source: Front Psychol. 2022 Jul 15;13:857372. doi: 10.3389/fpsyg.2022.857372 (PMC9335152; doi:10.3389/fpsyg.2022.857372)
Supplement: Supplementary file 1 [file Table_1.DOCX]

**Supplementary material.**

  To see how stimuli in the environment (e.g., sounds, noise, light, cold etc.) and natural elements (e.g., water, animals etc.) affected the level of mystical and or challenging experiences, correlations were calculated between these elements and the scores on MEQ and CEQ total scores. These are displayed in table 1.

 Separate correlations were calculated between MEQ/CEQ ratings and stimuli that were experienced as bothersome (1 = did not bother me, 4 = bothered me a lot) and natural elements (0 = not present, 1 = present). Overall, the scores on bothersome stimuli were low (Mean ranging between 1.03 and 1.56) and did not affect the level of mystical experiences. Bothersome stimuli were correlated to challenging experiences in that the more bothersome the stimuli were experienced, the more challenging the experience was (see table 1). For natural elements, only fire and animals were related to MEQ and CEQ. The presense of fire was related to more mystical and more challenging experiences. The same was true for the presence of animals (see table 1).

**Table 1**. Correlations between the MEQ/CEQ and bothersome elements and natural elements by tradition. ** p < .001.

| *Bothersome elements* | *MEQ* | *CEQ* | *Natural elements* | *MEQ* | *CEQ* |
| --- | --- | --- | --- | --- | --- |
| Sound |  | .191** | Fire | -.220** | -.205** |
| Smell |  | .212** | Water |  |  |
| Brightness |  |  | Rain |  |  |
| Darkness |  | .139** | Plants |  |  |
| Noises |  | .217** | Natural grounds |  |  |
| Heat |  | .137** | Animals | -.118** | -.142** |
| Cold |  | .176** |  |  |  |
| Colors |  | .155** |  |  |  |
| Insects |  |  |  |  |  |
